# May the analysis of 1918 influenza pandemic give hints to imagine the possible magnitude of Corona Virus Disease-2019 (COVID-19)?

**DOI:** 10.1186/s12967-020-02673-6

**Published:** 2020-12-22

**Authors:** Raffaele Scarpa, Francesco Caso, Luisa Costa, Saverio Passavanti, Maria Grazia Vitale, Claudia Trojaniello, Antonio Del Puente, Paolo A. Ascierto

**Affiliations:** 1 Rheumatology Unit, Department of Clinical Medicine and Surgery, University of Naples Federico II, Via Sergio, Pansini 5, 80131 Naples, Italy; 2 Department of Melanoma, Cancer Immunotherapy and Development Therapeutics, Istituto Nazionale Tumori IRCCS Fondazione Pascale, Naples, Italy

**Keywords:** COVID-19, Immune response, JAK-inhibitors, Monoclonal antibodies, Passive antibody administration, SARS-CoV-2, Spanish influenza, Antiviral therapy, Biologics, Vaccines

## Abstract

**Background:**

In 1918 an unknown infectious agent spread around the world infecting over one-third of the general population and killing almost 50 million people. Many countries were at war, the First World War. Since Spain was a neutral country and Spanish press could report about the infection without censorship, this condition is commonly remembered as “Spanish influenza”. This review examines several aspects during the 1918 influenza pandemic to bring out evidences which might be useful to imagine the possible magnitude of the present coronavirus disease 2019 (COVID-19).

**Methods:**

In the first part of this review we will examine the origin of the SARS-Coronavirus-2 and 1918 Spanish Influenza Virus and the role played by host and environment in its diffusion. We will also include in our analysis an evaluation of different approaches utilized to restrain the spread of pandemic and to treat infected patients. In the second part, we will try to imagine the magnitude of the present COVID-19 pandemic and the possible measures able to restrain in the present environment its spread.

**Results:**

Several factors characterize the outcome in a viral pandemic infection. They include the complete knowledge of the virus, the complete knowledge of the host and of the environment where the host lives and the pandemic develops.

**Conclusion:**

By comparing the situation seen in 1918 with the current one, we are now in a more favourable position. The experience of the past teaches us that their success is linked to a rapid, constant and lasting application. Then, rather than coercion, awareness of the need to observe such prevention measures works better.

## Background

In 1918, an unknown infectious agent spread around the world infecting over one-third of the general population and killing almost 50 million people. Many countries were at war, the First World War. Since Spain was a neutral country and Spanish press could report about the infection without censorship, this condition is commonly remembered as “Spanish influenza”. Instead, it has been widely recognized that the infection started in the United States of America (USA), in military camps of the Kansas, spreading later in Europe and in the rest of the world after the arrival of American troops in France [1–3].

In the first part of this review we will examine the origin of the virus and the role played by host and environment in its diffusion. We will also include in our analysis an evaluation of different approaches utilized to restrain the spread of pandemic and to treat infected patients.

In the second part, we will try to imagine the magnitude of the present COVID-19 pandemic and the possible measures able to restrain in the present environment its spread.

## Influenza pandemic

The recent work by Short and coworkers well highlights several aspects about the origin of the 1918 Influenza Pandemic. There is definite evidence that 1918 influenza pandemic started in the USA, then it spread to Europe and also to the rest of the World, with the movements of American troops [1, 2]. The first American outbreak of 1918 influenza pandemic is usually reported at Camp Funston, an Army training camp located at Fort Riley, southwest of Manhattan in Kansas. However, in late January and early February in Haskell County, in the state of Texas, at three hundred miles west of Funston, a particularly aggressive influenza had been already observed [3]. In fact, local press reported about many people hit by the influenza epidemic, some progressing to pneumonia with several cases of death. We may assume that, because young people of Haskell had moved to Camp Funston for their military training, the outbreak exploded there, on March 1918, probably originated from that previously observed in Haskell. In a few weeks, several soldiers required hospitalization with treatment at the infirmaries scattered around the Army camp. Funston was a critical base for the American troop movements to other military camps and to Europe. Therefore, we may suppose that this was the road for the diffusion of the Influenza pandemic in other American Army bases and later on in France, particularly at Brest, the largest port of disembarkation for American soldiers in Europe [1–3].

The virus sustaining the 1918 Influenza pandemic was a type A, H1N1 subtype strain. Taubenberger and colleagues sequenced the entire 8-segment genome of the 1918 influenza virus, using RNA fragments recovered from the lungs of several victims. Sequence analysis suggests that the ultimate ancestral source of this virus is almost certainly avian [4, 5]. At least 2 different H1N1 influenza-virus strains circulated simultaneously in 1918. They had markedly different receptor-binding specificities (the first only for human/mammalian cells, the second for both mammalian and avian cells) and both were fatal to humans [6, 7]. At that time, when pandemic arrived, seasonal influenza viruses were not yet known. In fact their existence was demonstrated only in 1933 [7]. On the other hand, the discovery of the viral agent sustaining 1918 influenza pandemic was possible only in the late 1990s, when the viral genetic material was isolated from victims buried in Alaska permafrost [8, 9]. In addition, also original animal reservoir of the 1918 influenza virus remains still controversial. Two hypotheses have been advanced to explain the introduction of the virus in the human population. The first of these supposes a direct introduction from a single unidentified host [5]. The second sustained that 1918 Influenza virus originated from a reassortment process between avian, swine and/or human Influenza viruses in the years prior to the 1918 pandemic [10]. The absence of Influenza virus sequence data prior 1918 pandemic, leaves this question unanswered. In addition, 1918 influenza pandemic spread in three consecutive waves occurring in the 1918 spring, in the 1918 autumn (the worst) and in the 1918-1919 winter. Analysing the mutations in Haemagglutinins (HA) sequences of viruses circulating in the spring and in the autumn waves, an increased mutation of the HA sequences has been found in the virus sustaining the second. This phenomenon dramatically increased the ability of virus to binding the human receptors of the cells of the respiratory tract [1].

## The role played by the host

The importance of the role played by the host in 1918 influenza pandemic is supported by the different outcomes that infection had both within and among different populations. Several individual aspects sustain this idea. The most important include the age of infected subjects, their humoral and cellular immune response and their metabolic profile. Our review will analyse these points.

Usually, in influenza pandemic older subjects are at increased risk of developing fatal outcome. In 1918 instead young adults exhibited a high mortality rate. This point is still not fully clarified, although problems related to the immune status of the host are supposed [1].

In 1918 influenza pandemic, subjects born before 1889 {aged 30-60 years) showed a better outcome when compared to younger people [11]. This intriguing response may be probably explained by the fact that older subjects had acquired cross-protective antibodies having met in their life an H1 and/or N1 influenza virus and surely, in the period of time from 1889 to 1892, the H3 influenza virus which sustained the Russian Influenza pandemic [12]. In contrast, younger people born after 1889 were more immunologically naïve to influenza viruses. This aspect could have determined the lack of pre-existing cross-reactive antibodies, contributing to the high attack rate and the rapid spread of virus in the lower age groups. In addition, the immunologic vulnerability of young people may be also explained with a possible deficiency of cellular immunity determined by a defective response of CD8 + Tcells. This could be related to measles epidemics which were frequently described in the American military camps in the winter of 1917–1918. Young people infected by measles before 1918 were more susceptible to a severe influenza during 1918 pandemic [1, 13].

Malnutrition may reduce the immune response of the host to influenza viruses. Another related subject may be represented by famine which may increase the severity of all infections and also of influenza. In fact, high mortality rates in India during 1918 pandemic confirm this hypothesis [14]. On the other hand, overweight and particularly obesity may impair humoral and cellular immunity. A clear association between obesity and poor outcome in viral respiratory infections, such as influenza A (H1N1) infection has been reported. This point has been demonstrated in obese subjects who show a defective response of CD8 + T cells or a reduced production of antibodies after the seasonal influenza vaccine [15].

Further, in obese subjects, White Adipose Tissue (WAT) leads to a chronic inflammatory status due to upregulation of proinflammatory cytokines, with IL-1β and IL-6 and other adipokines representing main mediators [16].

A possible impact of obesity on inflammatory status and respiratory tract infection prognosis could be due not only to the alteration of pulmonary physiology, but also to a concomitant WAT- mediated pro-inflammatory status [16].

## Approaches adopted to limit the spread of influenza virus in 1918 pandemic

### Maritime quarantine

During the 1918 influenza pandemic, maritime travels were the most common transport systems for both tourist and commercial purposes. Therefore, several countries adopted quarantine measures on incoming ships to restrain epidemic. These initiatives resulted often of limited value because introduced too late, or because unable to detected subjects infected but asymptomatic. However, in some cases, when correctly and promptly applied, they helped to protect populations from the worst of the pandemic, as in the case of Australia and of American Samoa [17, 18].

### Measures of social distancing and of individual prevention

In 1918, most American cities imposed restrictions on person-to-person relationships and schools, churches, theatres and generally common meeting places were closed. In addition, some mass gatherings such as wedding, funerals and conferences were prohibited. These measures resulted particularly effective when adopted early and when maintained for sufficient time. In fact, when relaxed, usually viral spread restarted. Individual prevention measures, including the use of facemasks and the use of hand sanitizers, showed controversial results. In fact, while the use of facemasks did not produce sure positive effects in the protection against contagion, in contrast, handwashing and the use of hand sanitizers had a clear protective effect. Recent evidence supports the idea that measures of social distancing added to those of individual prevention, introduced at an early phase and prolonged for a long time, showed the best results in the prevention of contagion. San Francisco, Saint Louis, Milwaukee and Kansas City, that prepared the most effective intervention reduced transmission rates by up to 30–50%. In addition, data supported the reduction of high levels of mortality when social distancing was effectively pursued [19, 20].

## Clinical manifestations

1918 Influenza pandemic developed through three waves [21]. Although it globally affected many young adults, the initial, the spring wave (March–June 1918), was clinically mild, without significant effects upon general mortality rates. The second, the autumn wave (late August–December 1918), was unbelievably aggressive with an enormous mortality peak in the healthy young adults. It presented with high fever, cyanosis, and pulmonary oedema. Usually, 7–10 days after the onset of symptoms, patients deceased. In a little percentage, death arrived more rapidly, within 72 h from the onset of symptoms. The third pandemic phase started in January 1919 and was surprisingly less aggressive. Autoptic examinations particularly performed during the second wave, revealed at lung sections two possible scenarios [22]. The first one, the most common, occurred in patients who had lived for many days after the onset of symptoms. It consisted of an acute aggressive diffuse broncho-pneumonitis with images of microvasculitis and tissue necrosis, haemorrhage and oedema, complicated by the presence of bacteria (including streptococcus pneumoniae, streptococcus pyogenes, haemophilus influenzae and staphylococcus aureus). The second, occurring in no more than 15 per cent of the total fatal cases, particularly those who had lived only few days after the onset of pulmonary signs, consisted of a severe and acute respiratory distress-like syndrome (ARDS) with lungs partially collapsed, dark red, relaxed, with pleural surfaces showing extravasations. On section, lungs appeared dark red and wet [22]. Bronchial lymphnodes were enlarged and dark red. These particular aspects were consistent with an aberrant inflammatory response to 1918 influenza pandemic virus. We may imagine an exaggerated release of proinflammatory cytokines mimicking a cytokine release syndrome (CRS) a condition which occurs when white blood cells and macrophages are activated and release inflammatory cytokines which further activate more leukocytes and macrophages [23]. In 1918 influenza pandemic, young people, those previously infected by measles, showed a CD8 + Tcell defective response which exposed subjects to the development of an aberrant inflammatory response amplifying the severity of influenza symptoms [13].

## The situation today

Today we are experiencing a pandemic from a new Coronavirus strain emerged in Wuhan, China, at the end of 2019 [24]. This new virus phylogenetically derives from Coronavirus that supports a Severe Acute Respiratory Syndrome (SARS-COV) and caused the outbreak in 2002 [25]. On February 11, 2020 the International Committee on Taxonomy of Viruses named this new virus as Coronavirus SARS CoV-2 [26] and the World Health Organization has named the disease caused by it as coronavirus disease 2019 (COVID-19) [27]. Although Coronaviruses usually sustain common colds, sometimes, when they pass from animal reservoirs to humans, they cause outbreaks [28, 29]. This is the case of the SARS-COV outbreak in China, in 2002 (reservoir the bats) [28] or the MERS-COV (Middle East Respiratory Syndrome-Coronavirus) outbreak in 2012 (reservoirs dromedary camels and/or bats) [29]. In the case of COVID-2019, several reservoirs have been suspected (bats, snakes, pangolins). Although the infection route of the first case remains unclear, the place where the contagion started has been probably identified in the Huanan Seafood Wholesale Market of Wuhan. COVID-19 likely spread out from China through air travels of possible infected travellers [24]. Although growing evidence sustain that SARS-COV-2 may be transmitted from asymptomatic people or with mild disease [26, 30, 31], its mortality rate appears lower than that reported in the case of SARS-COV or MERS-COV pandemics [32–34].

At this point of our investigation it would be interesting to ask which are the differences from our situation today when compared to the one already experienced by the humanity in 1918. Many aspects today are fortunately different since then. In 1918 there were no antibiotics for treating overlapping bacterial infections, there were no anti-inflammatory agents and there was neither the concept of intensive care nor anything that would come close to it in clinical practice. Even more the existence of the viruses was not even known and the knowledge of the molecular pathways of inflammation and proinflammatory molecules, such as cytokines, will only be demonstrated at the end of 1970s. So, by comparing the situation seen in 1918 with the current one, we are now in a more favourable position. COVID-19 could manifest in its most severe form with fever and pneumonia in up to 15% of COVID-19 cases, leading to Acute Respiratory Distress Syndrome (ARDS) in up to 5%, with a variable mortality rate in the different countries affected by the pandemic. This ARDS is believed to be caused by a cytokine release syndrome (CRS) and secondary hemophagocytic lymphohistiocytosis (sHLH), already observed in patients with SARS-COV and MERS-COV [35, 36] as well as in autoimmune/autoinflammatory diseases, leukemia and/or oncologic patients receiving Chimeric Antigen Receptor (CAR)-T cell therapy [37]. Both viruses use the Angiotensin-Converting Enzyme-Related Carboxypeptidase (ACE2) receptor to enter cells. This receptor is expressed on cardiopulmonary tissues and hematopoietic cells, particularly monocytes and macrophages [38]. The infection of monocytes, macrophages and dendritic cells by SARS-COV-2 activates and leads to secretion of IL-6 and other pro-inflammatory cytokines. IL-6 binds to membrane-bound IL-6 receptor (mIL-6R) in a complex with Glycoprotein 130 (gp130) [39]. Then, Janus Kinases (JAK) and (signal transducer and activator of transcription 3 (STAT3) mediate downstream signal transduction. JAKs mIL-6R is located on immune cells, whereas membrane-bound gp130 is more ubiquitous. Regarding cis signaling, subsequent effects on the acquired immune system (B and T cells) and innate immune system (neutrophils, macrophages and Natural Killer cells) can contribute to CRS. In the case of trans signaling, IL-6 binds to the soluble form of IL-6R (sIL-6R), leading to a complex with a gp130 dimer on potentially all cell surfaces [40]. Activation of the IL-6–sIL-6R–JAK–STAT3 signaling at level of endothelial cells induces a “cytokine storm”. Vascular Endothelial Growth Factor (VEGF), monocyte chemoattractant protein-1 (MCP-1), IL-8, additional IL-6 are also secreted by endothelial cells and E-cadherin expression is reduced [40]. These changes contribute to vascular permeability and leakage and to ARDS hypotension and pulmonary dysfunction pathophysiology. Macrophage Activation Syndrome (MAS) is a hyperinflammatory syndrome characterized by CRS, cytopenia and multiorgan failure in which IL-6 and ferritin level are particularly elevated. MAS is often triggered by severe viral infections but it also verifies in oncologic patients on CAR T cell therapy [41]. Moreover, more recent evidence report that COVID-19 may be also complicated by a coagulopathy linked to a disseminated intravascular coagulation which results in a thrombotic and/or thromboembolic disease [42–44]. We have to consider that the development of thrombotic and thromboembolic disease could be a direct consequence of the systemic inflammatory process (thrombo-inflammation) [45–48]. In particular, it has been demonstrated that elevated IL-17A levels strongly correlates with vascular dysfunction in subjects affected by rheumatoid arthritis [49]. It has also been reported that high IL-17A levels increase, in both mouse and human, platelet activation [50] and modulate, in vivo, arterial thrombus formation [51] through the extracellular signal-regulated kinase-2 (ERK-2) signaling pathway [52]. In addition, a recent study reports that IL-17A promotes deep vein thrombosis in humans and mice by enhancing platelet activation/aggregation, neutrophil infiltration, and endothelial cell activation [53]. For these reasons, it could be possible that, in COVID-19 patients, IL-17A could potentially promote a pro-thrombotic state in the vascular system. Therefore, it could be useful to measure IL-17A levels in bronchoalveolar lavage fluid (BALF) and plasma/serum samples of moderate and severe COVID-19 patients [54].

## Therapeutic options

Differently from Spanish Influenza period, today, there are available many different therapeutic strategies for the management of COVID-19 patients.

Antiviral therapy provides nucleoside Analogs already approved for treatment of some viral infections (Favipiravir, Ribavirin, Remdesivir) [55–63], and Protease inhibitors (Lopinavir and ritonavir) [64, 65].

The two immunomodulatory agents, Chloroquine and hydrossicloroquine have shown in vitro an antiviral activity against SARS, MERS, HIV and Ebola through the inhibition of endosomal acidification [66–69]. After the recent demonstration in vitro of an efficacy also against COVID-19 [56], the two drugs, and particularly hydrossicloroquine showing the most potent antiviral effect, were suggested, alone and in combination with azithromycin, for COVID-19 patients. The use of this agents is still debated. [70, 71].

The treatment in Intensive Care Units (ICU) is reserved to COVID-2019 patients experiencing an aberrant immune response to viral infection resulting in an inflammatory cytokine storm and at risk of lethal outcome. Autoptic evidence have showed alveolar damage with oedema, proteinaceous exudate, focal reactive hyperplasia of pneumocytes with inflammatory infiltration of patchy and multinucleated giant cells [72, 73] and massive intravascular thrombosis of vessels of alveolar septi [74]. In ARDS, although steroids at high doses alone or associated to heparin and to antibiotics should represent an effective treatment for lung inflammation, controversial data in the literature seem to call everything into question [75]. In fact, steroids, alongside a definitely effective anti-inflammatory action, inhibit the immune response to the virus thus preventing its effective clearance [76]. In addition, the occurrence of adverse events are widely described at high and prolonged doses [77, 78]. More recently, the randomized control trial RECOVERY showed that Dexamethasone reduced deaths by one-third in ventilated patients (rate ratio 0.65 [95% confidence interval 0.48 to 0.88]; p = 0.0003) and by one-fifth in other patients receiving oxygen only (0.80 [0.67 to 0.96]; p = 0.0021). [79].

Therefore, the direct and rapid inhibition of molecules sustaining the inflammatory processes should assure a rapid and more effective stop of cytokine storm [80, 81]. Finally, the performance of different therapies used to treat ICU patients seems to be also depending by the clinical phase when subjects are treated. [82].

Tocilizumab is a humanized monoclonal antibody developed for blocking IL-6 receptors and has proved to be effective and safe in the treatment of patients with rheumatoid arthritis. Since some oncologic patients, when treated with CART-T cells therapies, may develop a CRS which may be stopped by IL-6 blocker, its use has been hypothesized to stop the cytokinic storm seen in COVID-19 patients [83]. Therefore, patients with severe or critical COVID-19 were recruited and given Tocilizumab according to standard treatment in which IL-6R inhibitor is associated to lopinavir, methylprednisolone, symptomatics and oxygen therapy [83]. Temperature of patients returned normal very quickly with a marked improvement of respiratory function and 20 patients, after two weeks were discharged. On March 19, 2020 the Italian Pharmaceutical Agency (AIFA) approved a Phase II trial where Tocilizumab was administered to 330 COVID-19 induced ARDS patients. By preliminary results, tocilizumab seems a promising approach in these severe cases [83].

These data are confirmed by several retrospective series of patients with severe COVID 19 pneumonia treated with tocilizumab. In these case series, tocilizumab showed meaningful activity in reduction of invasive mechanical ventilation and death [84–86]. However, a press release of the randomised, double blind, phase III trial COVACTA, which compared tocilizumab versus a matching placebo combined with standard of care, reported no statistically significant improvement in clinical status of patients with severe COVID 19 pneumonia. [87] In this study, no baseline IL-6 value, CPR and other inflammation markers were reported neither were used to select patients for the treatment. Actually, other two phase III trials EMPACTA and REMDACTA are evaluating the efficacy and safety of tocilizumab in combination with standard of care and in combination with remdesivir (NCT04409262, NCT04372186) [88]. More in general, no biomarkers data (like baseline IL-6 value, CPR, absolute lymphocyte count [ALC], neutrophil/lymphocyte ratio, ferritin, LDH, etc.) are available from phase II and phase III trials. Biomarkers exploratory analysis was reported in two different retrospective case series with tocilizumab and sarilumab (another anti-IL6 receptor). In these reports, baseline high level of IL-6 and neutrophil/lymphocyte ratio, a rapid decrease in CRP levels, and a rapid increase of ALC are related to response to tocilizumab as well as sarilumab. [89, 90].

Other promising therapeutic approaches provide IL-1 blockade by anakinra in COVID-19 patients developing ARDS, managed with non-invasive ventilation outside of the ICU [91] and JAK inhibition by Baricitinib [92].

In addition, given the possible role played by IL17 in the pathogenesis of massive intravascular thrombosis, the use of an anti-IL-17A neutralizing monoclonal antibody should be also proposed as a potential strategy [52].

## Vaccines

Vaccines by reducing morbidity and mortality, are the most effective strategy for the prevention of infectious diseases. Although, in the case of Coronavirus, at present, there are not approved vaccines, against COVID-19 more projects are ongoing, using various approaches. The spike protein of Coronaviruses is the most important target for the development of a vaccine. Its block interferes with the mechanism through which viral receptors bind to host cells (ACE2, APN and DPP4 receptors). At present several approaches for the development of vaccines are used including techniques for recombinant subunit vaccines, DNA vaccines or mRNA vaccines [93–96].

### Recombinant subunit vaccine

Subunit vaccine incites immune system without the introduction of infectious virus and shows an interesting safety profile [97]. They stimulate in vivo the response of T cells and the production of high titers of neutralizing antibodies [98, 99]. Studies are ongoing for the production of subunit vaccines against COVID-19. Preliminary results seem promising [100–102].

### DNA vaccine

DNA vaccine are an innovative approach for preventive or therapeutic purposes. They consist in direct injection of DNA plasmids expressing virus spike which induce the activation of T cells and stimulate a wide range of immune responses [103]. At present, pre-clinical trials are ongoing for the production of DNA vaccine against COVID-19 [104–106].

### mRNA vaccine

mRNA vaccines contain mRNAs encoding the antigens which were inoculated in the host by vaccination [107, 108]. mRNA vaccines represent an improvement when compared to conventional ones [108]. Their rapid development along with their intrinsic structural characteristics offer useful advantages. At present, a trial using a mRNA vaccine encoding viral spike protein of COVID-19 is ongoing [108].

## Passive antibody administration

In the absence of vaccines and/or monoclonal antibodies and/or effective drug therapies for COVID-19 patients, the use of plasma/sera of convalescent patients should be considered for providing immediate immunity to susceptible people. In 1918 influenza pandemic this therapeutic approach reduced the mortality of treated patients [109–114]. Recently the FDA ruled that the use of sera of convalescent patients should be routinely admitted only after the demonstration with clinical trials of their safety and efficacy. Therefore, the FDA, while not ruling out the possible use of this therapeutic approach in particularly compromised prognosis cases, has spurred the creation of a panel of experts for the development of an implementation protocol to rationalize the use of passive antibody administration [115].

## Monoclonal antibodies

Neutralizing monoclonal antibodies (mAbs) isolated from memory B cells of convalescent patients may be useful in order to treat SARS-CoV-2 infections due to the possibility of their production on a large scale and due to their therapeutic effectiveness. Infact, mAbs have shown both prophylactic and therapeutic efficacy against other infectious diseases such as HIV, Ebola and MERS [116–118] and their safety and potency in patients have been demonstrated in multiple clinical trials [119, 120].

These mAbs acts by binding to the ACE2 receptor on the host cells, which is used by the SARS-CoV2 to gain access to the cell through the spike (S) glycoprotein expressed on its surface [121]. The S1 subunit is responsible for virus attachment and contains the receptor-binding domain (RBD) which directly binds to the ACE2 receptor on the host cell while the S2 subunit mediates membrane fusion [122].

Three mAbs have been studied with really good results in mice expressing the humanized ACE2 (hACE2) receptor: BD-368-2 and B38 and H4; CB6, on the other hand, has been studied with other excellents results in vivo in rhesus macaque [123–125].

Another mAb against COVID-19, LY-CoV555 by Eli Lilly, has been jsubjected to a randomized, placebo controlled, double-blind, single-dose clinical trial which included 452 COVID-19 patients. Although only a phase II study, the authors concluded that LY-COV555 is both safe and effective in reducing the viral load and the percentage of patients who were hospitalized, compared to the placebo group [126, 127].

## Measures of public health intervention

Quarantine has been demonstrated to be effective in 1918 influenza pandemic. In fact, when firmly imposed in Australia, it protected people from the devastating effects of the second wave of 1918 influenza pandemic [17]. In addition, only the strict quarantine imposed by the Governor of the American Samoa prevented there the spread of the 1918 influenza pandemic. In contrast, this did not happen in western Samoa which was overwhelmed by the contagion brought by New Zealand ships [18]. Today maritime navigation is largely replaced by air navigation. This makes easier for pandemics to spread. It is therefore necessary to implement screening measures at airports at arrivals. Routes most at risk of spreading the pandemic should also be identified and information campaigns on the risks of travel for people who have had contacts with positive suspected should be implemented. Intervention strategies for restriction of mass gatherings are also of fundamental importance for the containment of contagion. It has been proved by the utility of lockdown in the Imperial study, in which the research assessed the impact of restrictions in 11 European countries-Austria, Belgium, Denmark, France, Germany, Italy, Norway, Spain, Sweden, Switzerland and the UK-up to the beginning of May. By that time, around 130,000 people had died from coronavirus in those countries. The researchers used disease modelling to predict how many deaths there would have been if lockdown had not happened. And the work comes from the same group that guided the UK’s decision to go into lockdown. They estimated 3.2 million people would have died by 4 May if not for measures such as closing businesses and telling people to stay at home. That meant lockdown saved around 3.1 million lives, including 470,000 in the UK, 690,000 in France and 630,000 in Italy [128]. Among these, school closure showed a documented utility. We may remember here the dramatic increase of 2009 influenza pandemic in Mexico when school activities started again [129]. The efficacy of intervention strategies is also dependent by their duration. The facemasks were a credible measure of infection prevention during the 1918 pandemic. Their use was particularly supported by the fact that they counteracted the possible transmission of the infection by air. In several U.S. cities, their use was made mandatory by law, and this provision was supported by pressing information campaigns [130, 131]. Nevertheless, a recent meta-analysis supports physical distancing of 1 m or more and optimum use of face masks, respirators, and eye protection prevent the spread of the SARS-Cov 2 [129]. Their use is extremely important for medical personal who could be directly exposed to the possible contagion when assisting patients [132, 133].

Finally, Spanish influenza teach us of pay close attention to factors contributing to the generation of its multiple waves incidence mainly represented by schools opening and closing, and changes in human behaviour in response to the outbreak [134].

## Conclusions

The experience of the past teaches us that battle against pandemic is linked to a rapid, constant, and lasting application. Fortunately, today, knowledge in medicine, particularly in the field of therapies and vaccination are greatly improved when compared to the last century. This is also the case of most sophisticated laboratory advancement able for example to better characterize genotype host characteristics, such as Human leukocyte antigens (HLAs) polymorphisms, involved often in virus susceptibilities.

HLAs are proteins encoded by a several human genes located in the major histocompatibility complex and recognize infectious stimuli leading to immune defense against infection. These can show heterogenous and differ for ethnicity and geographic distributions. HLA variation affects the cellular immune response to coronaviruses peptides and HLA type of patients affected by COVID-19 has been called in cause in addressing disease severity and clinical outcome [135]. So, by comparing the situation seen in 1918 with the current one, we are now in a more favourable position. Then, rather than coercion, awareness of the need to observe such prevention measures works better. A future mass vaccination campaign against COVID-19, as well other infectious diseases involving administration of vaccine doses to a large population over a short period of time is expected [136]. Very effective for this purpose are information campaigns that sometimes involve well-known figures, especially from the world of entertainment or sports.

The COVID-19 pandemic, which probably is the most dreadful one in the last 100 years after Spanish flu, has posed a difficult challenge for any individual. However, differently from Spanish Influenza, today, more sophisticated measures of Public Health Intervention have led to better strategies for fighting the virus and reducing the impact of negative health social, and economic pandemic effects [137]. At first, the COVID-19 pandemic has addressed the speedy use of multiple approaches acting on pathogenesis of this SARS-COV-2, by immunomodulants, monoclonal antibodies and antivirals drugs. COVID-19 pandemic has urged the scientific experts to find rapidly responses in terms of vaccines to control SARS-CoV-2 [137]. Not casually, COVID-19 represents the only outbreak to date in which in a time of less than 9 months from pandemic emergence, there has been a mounting development of vaccines [138]. Unfortunately, it remains an imperative request for vaccine development since no effective therapies or vaccines have been approved to date [138]. Further, it is still far too early to know what the best vaccines will be to prevent or minimize COVID‐19 and their approval will depend upon the results of the efficacy and safety studies [138]. Of note, today we know that effective interventions to reduce the spread of the virus are represented by more testing, wearing face masks, and social distances [138].

As healthcare systems are very focused on COVID-19 care, the spread of the SARS-COV-2 also carry negative health effects, and an example is represented by limitation of access for chronic and not-surge diseases [139, 140]. Economic effects cannot be separated from health effects, and interventions designed to control COVID-19 need to take account of consequences [141].

It is today clear that as there is a need for rapid, innovative, and cost-effective emergency response mechanisms and the presence of gaps in critical care volume come to be conspicuously evident in most countries worldwide [142]. To respond to the growing demand of ICU beds and ventilators, frameworks for rationing have been developed to ensure the reasonable allocation of scarce resources [143]. Worldwide, the exponential increase in COVID-19 cases and subsequent demand for ICU beds is overcoming the capacity of even the largest hospitals. It is need for this pandemic that Countries maintain measures to strictly control the rate of new cases and continue to improve ICU bed capacity [144]. Additionally to the increase of ICU bed capacity, it is key that national health service provide a large increase in invasive ventilators and clinician and nursing staff numbers [145].

## Data Availability

All data generated or analyzed during this study are included in this published article.

## Abbreviations

ACE2: Angiotensin-Converting Enzyme
ARDS: Acute respiratory distress-like syndrome
BALF: Bronchoalveolar lavage fluid
CAR: Chimeric Antigen Receptor
COVID-19: Coronavirus disease 2019
CRS: Cytokine release syndrome
H1N1: Hemagglutinin 1 and Neuraminidase 1 subtype
ICU: Intensive Care Units
IL1: Interleukin-1
IL-6: Interleukin-6
IL-17A: Interleukin-17A
JAKs: Janus Kinases
mAbs: Monoclonal antibodies
MAS: Macrophage Activation Syndrome
MERS-COV: Middle East Respiratory Syndrome-Coronavirus
mIL-6R: Membrane-bound IL-6 receptor
NAK: Numb-associated kinase
SARS-COV-2: Severe Acute Respiratory Syndrome Coronavirus -2
sHLH: Secondary hemophagocytic lymphohistiocytosis
STAT3: Signal transducer and activator of transcription 3
WAT: White Adipose Tissue
